# Cytokine regulation of immune tolerance

**DOI:** 10.4103/2321-3868.124771

**Published:** 2014-01-26

**Authors:** Jie Wu, Aini Xie, Wenhao Chen

**Affiliations:** 1 Department of Surgery, Center for Immunobiology and Transplantation Research, Houston Methodist Research Institute, Houston Methodist Hospital, Houston, Texas USA; 2 Center for Immunobiology and Transplantation Research, Houston Methodist Research Institute, 6670 Bertner Avenue, R7-216, Houston, Texas 77030 USA

**Keywords:** Cytokine, immune tolerance, T cell differentiation, regulatory T cell

## Abstract

The immune system provides defenses against invading pathogens while maintaining immune tolerance to self-antigens. This immune homeostasis is harmonized by the direct interactions between immune cells and the cytokine environment in which immune cells develop and function. Herein, we discuss three non-redundant paradigms by which cytokines maintain or break immune tolerance. We firstly describe how anti-inflammatory cytokines exert direct inhibitory effects on immune cells to enforce immune tolerance, followed by discussing other cytokines that maintain immune tolerance through inducing CD4^+^Foxp3^+^ regulatory T cells (Tregs), which negatively control immune cells. Interleukin (IL)-2 is the most potent cytokine in promoting the development and survival of Tregs, thereby mediating immune tolerance. IL-35 is mainly produced by Tregs, but its biology function remains to be defined. Finally, we discuss the actions of proinflammatory cytokines that breach immune tolerance and induce autoimmunity, which include IL-7, IL-12, IL-21, and IL-23. Recent genetic studies have revealed the role of these cytokines (or their cognate receptors) in susceptibility to autoimmune diseases. Taken together, we highlight in this review the cytokine regulation of immune tolerance, which will help in further understanding of human diseases that are caused by dysregulated immune system.

## Introduction

Studies of immune tolerance have generally centered around “self ” versus “nonself ” recognition. In other words, T and B cells of the immune system recognize specific nonself-antigens derived from invading pathogens and defend the host from infectious diseases. T and B cells that recognize self-antigens are often believed to be eliminated or become nonfunctional. Nonetheless, in the context of T cell development in thymus, the nearly-random V(D)J recombination of αβ-T cell receptor (TCR) gene segments unavoidably creates a repertoire of major histocompatibility complex (MHC)-restricted TCRs that potentially recognize self-antigens. The thymocytes that have a TCR potentially recognizing self-antigens undergo apoptosis, a process called negative selection or central tolerance. However, some self-reactive thymocytes successfully develop into mature T cells and enter the periphery. To ensure immune tolerance to self, the self-reactive T cells entering the periphery can be further deleted, become anergic (a lack of reaction against specific antigen), or be actively suppressed by regulatory T cells (Tregs). The molecular basis underlying these peripheral tolerogenic mechanisms is not fully defined.[1]

**Table 1:** 

Access this article online	
**Quick Response Code:**	**Website:** www.burnstrauma.com
	**DOI:** 10.4103/2321-3868.124771

As a hallmark of the adaptive immune system, T cells require antigen-specific activation to exert their effector function. This antigen-specific activation occurs when the T cells receive sufficient signals upon engagement of the TCR by the antigen-MHC complex on the antigen presenting cells (APC) in combination with signals from CD28-B7 co-stimulatory molecules.[2] Activated T cells in turn express high affinity interleukin (IL)-2 receptor (IL-2R) and also produce cytokine IL-2 themselves. The IL-2 signaling pathway drives the proliferative machinery of T cells.[3] To date, in addition to CD28-B7 and IL-2, numerous cell surface co-signaling molecules and soluble cytokines have been shown to control T cell activation and function.[2] Hence, T cell immunity or tolerance to a particular antigen is regulated not only by TCR recognition, but also by various co-stimulatory and cytokine signals. This is exemplified in mice deficient in cytotoxic T-lymphocyte antigen 4 (CTLA4), a negative regulator that turns off the CD28-B7 signaling, where they develop lethal autoimmunity in weeks after birth.[4] In this review, we focus on the cytokine regulation of immune tolerance, with specific emphasis on the peripheral tolerance of T cells, as they are the central players in various human autoimmune diseases.

## Cytokines that maintain immune tolerance

The cytokine milieu provides not only proinflammatory but also anti-inflammatory signals to control immune homeostasis and responses. Results from experimental works have revealed that mice deficient in immunosuppressive cytokines are prone to autoimmunity. Defining the biological functions of those cytokines (e.g., transforming growth factor (TGF)-b, IL-10, IL-27, and IL-37) will advance our understanding of immune tolerance and autoimmune diseases.[5]

### TGF-β

TGF-β has three mammalian isoforms, including TGF-β 1, TGF-β2, and TGF-β3. Among them, TGF-β1 is uniquely required for establishing and maintaining normal immune homeostasis,[6] which is evident by the fact that TGF-β1-deficient mice exhibit severe and lethal multiorgan inflammatory diseases shortly after birth.[7] Because of this, the inhibitory effects of TGF-β1 on various immune cell subsets have been extensively investigated. Indeed, TGF-β1 inhibits the maturation and antigen presentation of dendritic cells (DCs) and macrophages, reduces the interferon (IFN)-γ production and the cytotoxic activity of natural killer (NK) cells, modulates the differentiation/proliferation and IgA production of B cells, and constrains the differentiation/proliferation and perforin/Fas ligand expression of CD8^+^ cytotoxic T lymphocytes (CTL).[8]

It becomes interesting as to whether the early lethal autoimmunity observed in TGF-β1-deficient mice is attributed to the dysregulation of T cells, as these cells play a central role in breaking and maintaining immune tolerance. Two independent groups addressed this question by crossing the TGFβRII-flox/flox with CD4-Cre mice, which leads to the disruption of TGF-β signals only in T cells.[9,10] They found that TGF-βRII deficiency in T cells results in the generation of highly pathogenic T cell subsets with overexpressed FasL, perforin, granzymes, and IFN-g, which in turn causes early-onset lethal autoimmunity. Hence, TGF-β1 control immune homeostasis mainly, if not entirely, through restraining T cell activation and differentiation. Indeed, TGF-β1 potently inhibits T cell activation by repressing the activation of Tec kinase Itk and calcium influx. Moreover, TGF-β1 diminishes T helper (Th)1 and Th2 cell differentiation by inhibiting the expression of the master regulators T-bet and GATA-3, respectively.[8]

Exogenous TGF-β1 is frequently used for the *in vitro* differentiation of both inducible Treg (iTreg) and Th17 cells. TGF-β1 is thus considered as an important regulator that controls the balance of Th17 and Tregs,[11] but the physiological role of this regulation *in vivo* is unclear. Taken together, TGF-β1 permits immune tolerance by modulating the activation and differentiation of immune cells, in particular T cells.

### IL-10

IL-10 is a cytokine with pleiotropic effects on many immune cells. For instance, IL-10 modulates the function of APCs through inhibiting phagocytosis, downregulating the expression of MHCs and co-stimulatory molecules, and decreasing the production of proinflammatory cytokines and chemokines.[12] Moreover, IL-10 directly inhibits the differentiation of Th cells and maintains the suppressive activity of Treg cells.[13]

IL-10-deficient mice spontaneously develop colitis,[14] suggesting that IL-10 exerts *in vivo* immunoregulatory effects largely in the intestinal tract. In particular, IL-10 produced by Tregs or Tr1 cells as well as IL-10 signaling in Tregs is believed to be required for preventing T-cell mediated colitis.[13,15,16] In human, IL-10 has also been confirmed as a susceptibility gene for inflammatory bowel disease (IBD).[17] The beneficial effects of IL-10-based therapies to treat IBD in the clinic remain to be determined.[18]

### IL-27

IL-27 is an IL-12 family cytokine composed of heterodimeric subunits p28 and Epstein-Barr virus-induced gene 3 (EBI3).[19] IL-27 is produced mainly by APCs. The immunoregulatory effects of IL-27 include suppressing Th17 cell differentiation,[20,21] facilitating Treg generation,[22] and promoting IL-10-mediated T cell tolerance.[23] Results from a recent study[24] indicate that IL-27 also directly acts on DCs themselves. IL-27 signaling induces immunosuppressive DCs to express high levels of CD39, which in turn promotes the differentiation of Tregs. IL-27 signaling in DCs also inhibits the differentiation of Th1 and Th17 cells, and prevents the development of experimental autoimmune encephalomyelitis.[24]

### IL-37

IL-37 (IL-1 family member 7) is a newly identified antiinflammatory cytokine affecting both innate and adaptive immunity.[5,25] IL-37 has five splice variants (IL-37a–e). Transgenic expression of IL-37 protects mice from lipopolysaccharide-induced shock and dextran sulfate sodium-induced colitis[26] as well as concanavalin A-induced hepatitis, [27] probably through inhibiting the production of pro-inflammatory cytokines IL-17 and tumor necrosis factor (TNF)-α.

## Cytokines that control Treg biology

CD4^+^Foxp3^+^ Tregs are indispensable in immune tolerance to self-tissues, which is evident by the fact that deficiency of a functional Foxp3, the master regulator of Treg development and function, leads to severe autoimmunity and early mortality in both humans and mice.[28] All T cell subsets require cytokine signals for survival and function, and Tregs are no exception. Elucidating the cytokines that control Treg biology will advance our understanding of immune tolerance as well as the therapeutic use of Tregs in autoimmune diseases.

### IL-2

IL-2 signals through its high-affinity IL-2R (consisting of the IL-2Rα (CD25), IL-2Rβ, and common γ-chain (γc) subunits) and is essential for the expansion of activated effector T (Teff) cells.[3] Therefore, it was somewhat unexpected that mice deficient in IL-2, IL-2Rα, or IL-2Rβ developed lethal autoimmune diseases. Indeed, the lethal autoimmunity observed in those mice are attributed to IL-2 signaling defect in Tregs, which constitutively express high-affinity IL-2R and utilize IL-2 as a survival factor.[3] Moreover, studies by our group and others have shown that Tregs exert their suppressive function at least partially through consuming IL-2, which creates an IL-2-deprivative environment to limit Teff cell expansion.[29]

The IL-2 signaling molecule, Stat5, can bind to the promoter region and an intronic regulatory deoxyribonucleic acid (DNA) element within the Foxp3 locus,[30] suggesting that IL-2 signaling also controls the generation of Foxp3 expressing Tregs. It is believed that Tregs are either derived from the thymus as natural Tregs (nTregs) or generated *de novo* from peripheral CD4^+^Foxp3- T cells as iTregs.[28] A “two-step model” of thymic nTreg development suggested that TCR-ligand interaction on CD4 single positive thymocytes results in the generation of CD4^+^CD25^+^Foxp3 nTreg precursors, followed by an IL-2-directed step that subsequently induces Foxp3 expression in these nTreg precursors.[31] We found that the two-step model holds true for iTreg development as well. IL-2 indeed is the most potent cytokine that induce Foxp3 expression in CD4^+^CD25^+^Foxp3- iTreg precursors. Nevertheless, at the initial TCR-directed phase, inhibition of IL-2 signaling is required for the generation of iTreg precursors. Thus, IL-2 plays a dual role in iTreg generation.[32]

We speculate, based on the two-step iTreg generation model, that when T cells receive suboptimal or chronic antigen (Ag) stimulation in the absence of robust IL-2 signaling, some of them develop into iTreg precursors and remain in the environment, where, over time, sufficient levels of IL-2 may become available to induce Foxp3 expression. In most *in vitro* studies, high amounts of exogenous TGF-β (at the nanogram per milliliter level) are used to generate iTregs from naive CD4^+^ T cells. Such high amounts of TGF-β may be rare under physiological conditions. In our two-step model, exogenous TGF-β is not required, but neutralizing TGF-β or blocking TGF-βR signals decreased iTreg generation. Thus, this two-step iTreg generation still is favored in unique environments where certain levels of TGF-β signals are available, such as GALTs, to drive Treg induction.[32]

The significant role of IL-2 played in Treg generation and homeostasis has motivated scientific research on developing IL-2-based immunotherapy for autoimmune diseases [33]. For instance, in nonobese diabetic (NOD) mice, insulin dependent diabetes susceptibility 3 (Idd3) is the strongest single non-MHC T1D susceptibility locus, which contains Il2 gene that transcribes much less IL-2 mRNA when compared to the Idd3/Il2 allele in T1D-protected mouse strains.[34] With the notion that IL-2 deficiency impairs Treg function, transient low-dose IL-2 was administrated in diabetic NOD mice to “correct” Treg function, which indeed partially reversed the T1D progression.[35] In human subjects, polymorphisms in genes transcribing IL-2Rα and IL-2 are strongly associated with T1D. In particular, individuals with IL2RA susceptibility genotypes is associated with lower expression of IL-2Rα on Tregs and lower IL-2 production by Teff cells, both of which may impair Foxp3 expression and Treg function.[34] Clinical trial of IL-2 administration in T1D subjects has thus been attempted. IL-2 therapy combined with rapamycin increased Treg frequency in T1D patients. However, this combined therapy cause transient beta-cell dysfunction rather than protection,[36] and the mechanism for this unexpected outcome remains to be defined.

Teff cells also express high affinity IL-2R upon TCR stimulation. IL-2 thus potently affects Teff differentiation (promoting Th1 and Th2 while inhibiting Th17 and follicular helper T cell (T)), expansion cytolytic activity, and development into functional memory cells.[3] Caution should be exercised for developing IL-2 therapy in autoimmune diseases. In particular, the balance between Teff cell responses and Treg-mediated tolerance must be taken into consideration in designing IL-2 based therapies. Nevertheless, Il-2 or IL2RA are genetic loci with confirmed associations with T1D, rheumatoid arthritis (RA), celiac disease, multiple sclerosis (MS), and Graves’disease (GD).[37] The unsuccessful trial of IL-2 therapy in T1D should not halt further research in understating IL-2-Treg pathway.

### IL-35

IL-35, which is composed of IL-12 subunit p35 (IL-12α) and IL-27 subunit β (Ebi3), is produced predominantly by Tregs and is required for their maximal suppressive activity.[38] IL-35 treatment *in vitro* can suppress T cell proliferation and convert naive T cells into IL-35-producing induced Tregs (iTr35 cells) that do not express Foxp3.[39] Surprisingly, neither Ebi3 ^-/-^ nor Il12a^-/-^ mice have overt autoimmunity or inflammatory diseases.[38] It is possible that the defect of IL-35 action in these mice is counterbalanced by the loss of inflammatory IL-12 family cytokines. The *in vivo* physiological role of IL-35 in immune tolerance remains to be better explained.

## Cytokines that break immune tolerance

T cell survival and function are supported by various cytokines (IL-7, IL-12, IL-21, IL-23, etc.). Recent genome-wide association studies (GWAS) firmly demonstrated the genetic association between genes coding for these cytokines (or their cognate receptors) and various human immune disorders. Cytokine blockade thus has therapeutic potential to halt T cell-mediated autoimmune diseases.

### IL-21

IL-21 is produced predominantly by CD4^+^ Teff cells (Th17, T_FH_, and other activated CD4^+^ cells) and NKT cells. The production of IL-21 by CD4^+^ Teff cells is mediated by IL-6, IL-21 itself, and International Code of Signals (ICOS), which activate the transcription factors Stat3, IRF4, and Batf. IL-21R plus the common γcform the functional IL-21 receptor, which is broadly expressed on B, T, NK, and DCs.[40] The binding of IL-21 with its receptor exerts pleiotropic effects on these immune cells. For instance, IL-21 is a polarizing cytokine for the differentiation of Th17 and T_FH_ cells, which in turn produce high amount of IL-21. IL-21-producing T_FH_ cells control the differentiation of germinal center B cells and immunoglobulin production.[40] Therefore, IL-21 block or IL-21R deficiency prevents B cell-mediated humoral immunity in lupus-prone mouse models.[41,42]

IL-21 and IL-2 are closely-related cytokines that share the common γc receptors. Both cytokines are predominantly produced by Th cells. Moreover, the genes encoding IL-21 and IL-2 are adjacent to one another.[33] In NOD mice, the Idd3 T1D susceptibility locus contains genes encoding for both of them. Although no functional variants of IL-21 has been identified in NOD mice, due to the critical role of IL-21 in T cell-mediated pathogenesis, IL-21 or IL-21R knockout renders NOD mice resistant to the onset of T1D.[43,44] In human, the IL-2/IL-21 locus is a common genetic factor in autoimmune diseases, including T1D, RA, and celiac diseases.[37] It is essential to determine the functional variants of both IL-2 and IL-21 that leads to the breach of immune tolerance to self-tissues.

### IL-12 and IL-23

IL-12 and IL-23 are proinflammatory cytokines among the four heterodimeric cytokines of IL-12 family. An IL-21B p40 subunit is shared by both cytokines, and it dimerizes either with p35 to form IL-12 or with p19 to form IL-23. Moreover, the receptors of both cytokines share the IL-12Rβ1, which combines with IL-12Rβ2 to form the receptor for IL-12 or with IL-23R to form the receptor for IL-23.[45]

Although both IL-12 and IL-23 are produced by APCs upon immune stimuli, they direct T cell response in a very different manner. IL-12 initiates Th1 cell response. Binding of IL-12 to its cognate receptor expressed on activating T cells results in activation of Stat4, expression of T-bet, and initial production of IFN-γ. In turn, IFN-γ provides positive feedback to increase T-bet expression and IFN-γ production.[46] In contrast, IL-23R is not readily expressed on activating T cells, as its expression depends on the activation of Stat3 and the presence of Th17 master regulator RORγt.[47] Therefore, IL-23 does not initiate Th17 cell differentiation, and mainly serves as a survival cytokine to maintain Th17 cell response.

In human, IL-12Rβ1 deficiency leads to recurrent and/or severe infections caused by Mycobacteria and Salmonellae that are poor pathogenic in healthy individuals.[48] This is not surprising, as IL-12Rβ1 deficiency impairs both Th1 and Th17 cell responses. Furthermore, polymorphisms in IL-23R have been confirmed to be causative or protective for Crohn’s disease, ulcerative colitis, inclosing spondylitis, and psoriasis. Polymorphisms in p40 cytokine subunit, shared by IL-12 and IL-23, also associate with Crohn’s disease and psoriasis.[37] Hence, deficiency in IL-12/IL-23 signals results in defective immunity against infection, while quantitative changes in IL-12/IL-23 signals protect or break self-tolerance.

### IL-7

IL-7 supports T cell development and provides essential survival signals for naive and memory T cells.[49] IL-7 receptor is a heterodimer of IL-7Rα and the common γc. Because polymorphisms in the IL-7Rα have been associated with increased risk for MS,[37] it would be interesting to uncover the mechanisms underlying IL-7’s role in autoimmunity and immune tolerance and whether IL-7 modulates T cell differentiation/function in addition to its activity as a survival factor.

## Conclusions

**Figure 1:**
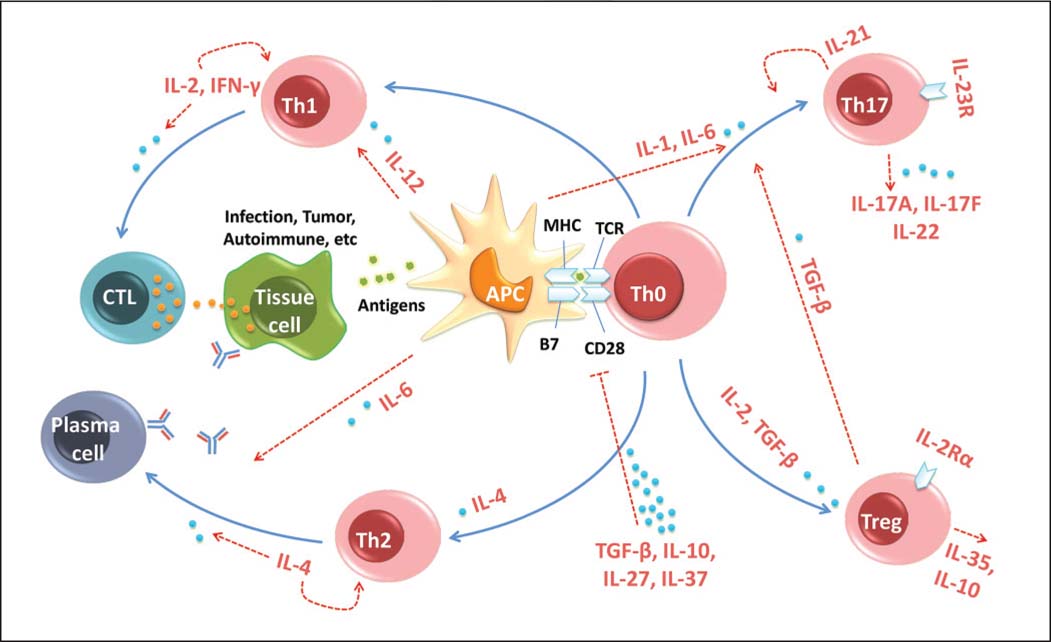
Cytokines regulate immune tolerance by affecting T cell activation and differentiation. Transforming growth factor (TGF)- β1, interleukin (IL)-10, and IL-27 inhibit the antigen presentation of antigen presenting cells (APCs) and alter the subsequent activation and differentiation of T helper (Th) cells. IL-2 is a tolerogenic cytokine that controls the generation and survival of regulatory T cells (Tregs). IL-21 serves as an autocrine cytokine in Th17 and follicular helper T cell (T_FH_) cell differentiation. IL-12 initiates Th1 cell differentiation, whereas IL-23 maintains Th17 cell differentiation. These cytokines are either required for maintaining self-immune tolerance or involved in autoimmune pathology.

It is well-recognized that not all self-reactive T cells are eliminated in the thymus. A traditional viewpoint suggests that peripheral tolerogenic mechanisms (e.g., T cell anergy, exhaustion, deletion, or Treg suppression) must exist in healthy individuals to disarm the remaining self-reactive T cells. In this review, we illustrate a different viewpoint of peripheral tolerance. We believe that cytokines affect T cell activation and differentiation, and in turn regulate immunity and immune tolerance [Figure 1]. The same should hold true for co-stimulatory molecules in the control of T cell activation. Recent advances in the genetics of autoimmune diseases indeed identify numerous risk molecules and genes that contribute to disease susceptibility. Further characterization of risk molecules and their expression and function *in vivo* will lead to better define the pathogenic pathways of human autoimmunity. This inquiry will also greatly aid in the induction of transplant tolerance.
